# Donkey-Hide Gelatin Peptide-Iron Complexes: Structural Characterization, Enhanced Iron Solubility Under Simulated Digestion, and Dual Iron Chelation-Antioxidant Functions

**DOI:** 10.3390/foods14122117

**Published:** 2025-06-17

**Authors:** Lili Yang, Chenyan Lv, Xingfeng Guo, Rong Liang

**Affiliations:** 1 Shandong Key Laboratory of Applied Technology for Protein and Peptide Drugs, School of Pharmaceutical Sciences and Food Engineering, Liaocheng University, Liaocheng 252059, China; 13931971560@163.com (L.Y.); guoxf0906@126.com (X.G.); 2 College of Food Science & Nutritional Engineering, China Agricultural University, Beijing 100083, China; 2019023@cau.edu.cn

**Keywords:** donkey-hide gelatin peptides, peptide-iron chelates, structural characterization, spectroscopy analysis, antioxidant activity

## Abstract

Iron deficiency is a global health issue, making the development of novel iron supplements to enhance iron absorption critically important. In this study, low molecular weight donkey-hide gelatin peptides (LMW DHGP) were enzymatically hydrolyzed from donkey-hide gelatin. Experimental results demonstrated that the iron chelating capacity of LMW DHGP reached 249.98 μg/mg. Key amino acids (Asn, Gly, Cys, Lys) may participate in chelation. Scanning electron microscopy (SEM) and X-ray diffraction (XRD) analysis showed rough, porous amorphous structures of LMW DHGP-iron complexes. The results of circular dichroism spectroscopy (CD) indicated that the self-assembly of LMW DHGP-iron complexes appears to be primarily mediated by peptide α-helical structural conformations. Fourier transform infrared (FTIR) spectroscopy further indicated that the interaction between LWM DHGP and Fe^2+^ likely occurs through carboxyl and amino functional groups. In vitro digestion stability studies demonstrated that LMW DHGP-iron complexes exhibited superior iron ion solubility compared to FeSO_4_ in simulated gastrointestinal conditions. PGPAG-iron complexes exhibited the highest antioxidant activity, with scavenging rates of 71.64% (DPPH radical) and 88.79% (ABTS radical). These findings collectively suggest that LMW DHGP-iron complexes possess significant potential as a novel iron supplement in food applications, which provides valuable theoretical insights for the development of innovative iron supplementation strategies.

## 1. Introduction

Iron is an essential micronutrient that is important in various biological processes, including hemoglobin and myoglobin synthesis, peroxidase activity, oxygen transport, hematopoiesis, immune function, energy production, and cellular proliferation. The human body primarily absorbs and utilizes two forms of iron: heme and non-heme iron, which are predominantly obtained from red meat and plant-based sources, respectively [1]. Non-heme iron exists in several forms, including soluble iron, low-molecular-weight iron complexes, storage iron in ferritin, and iron incorporated into the catalytic centers of various proteins. For effective absorption, these forms must be present in the intestinal lumen. In contrast, heme iron, which is encapsulated within the protoporphyrin ring, can be absorbed by intestinal mucosal cells directly. This structural configuration not only enhances its absorption efficiency but also provides protection against gastrointestinal irritation and reduces its susceptibility to dietary interference [2]. The absorption mechanism of non-heme iron is more complex, requiring its dissociation from organic ligands and conversion to ferrous ions (Fe^2+^) before mucosal uptake. This is because non-heme iron in food primarily exists as Fe(OH)_3_ complexes, which are not readily bioavailable. The bioavailability of non-heme iron is significantly influenced by various dietary factors and luminal conditions, making direct iron supplementation less effective compared to heme iron sources [3].

Iron deficiency remains a significant global health challenge, with iron loss or inadequate absorption potentially leading to adverse health consequences, including anemia, impaired energy metabolism, and compromised immune function. Given the limited bioavailability of iron from dietary sources, supplemental iron intake is often necessary [4]. The evolution of iron supplementation has progressed through three generations. The first generation, represented by inorganic iron supplements such as ferrous sulfate, is associated with gastrointestinal side effects and exhibits high variability in iron absorption, consequently affecting its bioavailability. Additionally, these supplements may induce undesirable alterations in food characteristics, including color, taste, and quality parameters [5]. The second generation of supplements, comprising ferrous organic salts (e.g., ferrous lactate), still presents non-negligible side effects, particularly on intestinal flora, and maintains relatively low absorption rates. In contrast, the third generation of iron supplements, specifically peptide-iron chelates, demonstrate superior characteristics, including high bioavailability, enhanced absorption efficiency, stability, safety, and nutritional value, making them a promising alternative for iron supplementation [6].

Currently, bioactive peptides derived from food proteins have garnered significant attention as novel metal ion chelators. Peptides are regarded as optimal ligands due to their possession of two functional groups capable of participating in iron coordination [7]. Shilpashree et al. [8] demonstrated that succinylation enhanced the solubility of milk protein concentrate, which, when complexed with iron, exhibited improved stability and potential as an organic iron source for various food applications. Furthermore, certain iron chelates have been shown to possess immunomodulatory effects [9]. Numerous studies have identified various peptide sources with iron-chelating capabilities, including casein [10], whey protein [11], abalone offal [12], red seaweed [13], wampee seed antioxidant peptides [14], and sea cucumber eggs [2], among others.

Donkey-hide gelatin, a traditional medicinal product derived from the concentrated boiling of donkey skin, is primarily composed of collagen and amino acids, with additional constituents including fatty acids, polysaccharides, and other bioactive compounds [15]. This substance possesses a broad spectrum of pharmacological activities, particularly exhibiting anti-inflammatory properties, hemostatic, anti-fatigue, immunomodulatory, antitumor, and anti-anemic effects. Its significant therapeutic benefits and high clinical value have been well-documented. In the food industry, donkey-hide gelatin has been innovatively incorporated into various nutritious products, such as donkey-hide gelatin cakes and porridges, which are not only palatable but also provide substantial health benefits. These products have gained considerable popularity among consumers due to their unique combination of nutritional value and sensory appeal [16].

Recent studies have demonstrated that donkey-hide gelatin can generate peptides with iron-chelating capabilities following gastrointestinal digestion, which facilitates intestinal iron absorption and ameliorates iron deficiency anemia [17]. Cheng et al. [18] reported that the relative iron bioavailability of donkey-hide gelatin-iron chelates and donkey-hide gelatin hydrolysate-iron chelates reached 123.45% and 117.70%, respectively, when compared to FeSO_4_ as a reference standard. These findings indicate that the chelated iron in donkey-hide gelatin-iron chelates and donkey-hide gelatin hydrolysate-iron chelates exhibits superior bioavailability compared to free iron in FeSO_4_. Notably, donkey-hide gelatin-iron chelates demonstrate enhanced chelating strength, attributed to its higher proportion of components with strong iron-chelating capacity. Despite the significant clinical value of donkey-hide gelatin and its increasing presence in the global market, research on the preparation of iron-chelating peptides derived from donkey-hide gelatin remains limited. Furthermore, the structural characterization, chelation mechanisms, and stability of these peptide-iron complexes are not yet fully elucidated, warranting further investigation.

In this study, we initially prepared low molecular weight donkey-hide gelatin peptides (LMW DHGP) through enzymatic hydrolysis, followed by the chelation of LMW DHGP with Fe^2+^ to synthesize LMW DHGP-iron complexes. The iron-chelating capacity of LMW DHGP was quantitatively assessed. Subsequently, comprehensive characterization of both LMW DHGP and LMW DHGP-iron complexes was conducted to identify the specific amino acids and functional groups involved in the chelation process. The alterations in surface morphology and secondary structure before and after chelation were systematically analyzed. Furthermore, the iron solubility of LMW DHGP-iron complexes and FeSO_4_ was assessed by in vitro gastrointestinal simulation assay. Finally, we proposed a mechanistic framework to elucidate how LMW DHGP-iron complexes facilitate enhanced iron absorption.

## 2. Materials and Methods

### 2.1. Materials and Reagents

Donkey-hide gelatin was purchased from Dong’E Shengliyuan Ejiao Co., Ltd. (Liaocheng, China). Potassium bromide (KBr), ascorbic acid, potassium persulfate (K_2_S_2_O_8_), formic acid, amino acid standard (AAS18), and acetonitrile were obtained from Sigma Chemicals Co. (St. Louis, MO, USA). Alcalase, porcine pepsin and trypsin were obtained from Novozymes Biotech. Co., Ltd. (CPH, Kastrup, Denmark). HCL, NaOH, trifluoroacetic acid, FeCl_2_·4H_2_O, acetic acid-sodium acetate buffer (pH 5.0), o-phenanthroline, bile salts, DPPH, ABTS, methanol, NaHCO_3_, FeSO_4_ were of analytical grade purity and obtained from Peking Chemical Plant (Beijing, China).

### 2.2. Preparation of Low Molecular Weight Donkey-Hide Gelatin Peptide (LMW DHGP) by Enzymolysis

The low LMW DHGP was synthesized following the protocol established by Kim et al. [19], with optimization of reaction conditions. A 4% donkey-hide gelatin solution was prepared and simmered in a water bath at 80 °C for 30 min. After cooling to the required enzymatic temperature of 63 °C, adjust the pH of the donkey-hide gelatin solution to 10.5. Then, Alcalase 2.4 L, enzyme to substrate ratio of 9%, was added to the solution for hydrolysis, the pH was maintained within ±0.05 during the enzymatic hydrolysis by adding NaOH or HCl solution as needed. After hydrolysis for 3 h, the enzyme was deactivated in a water bath at 90 °C for 10 min. Centrifuged (104 r/min) at 4 °C for 10 min. The supernatant was collected and pH was adjusted to 7.0. Finally, the LMW DHGP solutions were frozen and subsequently lyophilized at −50 °C for 24 h using a freeze dryer (Labconco, Kansas City, MO, USA).

### 2.3. Determination of Molecular Weight Distribution of LMW DHGP

Based on the method of Yan et al. [20] with methodological refinements, about 100 mg of sample was taken into a 10 mL volumetric flask, diluted to scale with mobile phase, sonicated for 5 min, centrifuged and then filtered through a microporous filter membrane and injected into the sample. Chromatographic column: TSKgel 2000 SWXL 300 mm × 7.8 mm; mobile phase: acetonitrile/water/trifluoroacetic acid, 40/60/0.1 (*v*/*v*); detection: UV220 nm; flow rate: 0.5 mL/min; column temperature: 30 °C; injection volume:10 μL; instrument: Waters 2695 high performance liquid chromatograph (with 2487 UV detector and Empower Workstation GPC software, version 3.6.1). Cytochrome C (MW12384), peptidase (Mw6511), mycopeptide (MW1422), ethionine-ethionine-tyrosine-arginine (MW451), ethionine-ethionine-Ethanine (MW189) were purchased from Sigma and used to make the standards used in the molecular weight calibration curve.

### 2.4. Determination of the Iron Ion Binding Capacity of LMW DHGP

Following the experimental protocol established by Wang et al. [21] after being slightly modified. The iron chelating ability of LMW DHGP was determined using the o-phenanthroline colorimetric method. The standard curve was plotted as follows: 10 μg/mL Fe^2+^ was used as the standard solution, and the gradient concentrations were set at 0, 0.40, 0.80, 1.20, 1.60 and 2.00 μg/mL. The Fe^2+^ solution was treated with 1 mL of 0.2 mol/L acetic acid-sodium acetate buffer (pH 5.0) and 200 μL of 20 mg/mL ascorbic acid, then mixed thoroughly. Add 5 mg/mL o-phenanthroline solution 400 μL to make the working color development solution, after 15 min of static reaction, using ultrapure water as a blank control, using a 96-well plate and enzyme labeling instrument to determine the absorbance value of the reaction solution at 510 nm, and then plotted according to the concentration of Fe^2+^ and the absorbance value of the standard curve.

Determination of ferric ion binding ability: 4% mass ratio of LMW DHGP solution was prepared first, i.e., 4 g of LMW DHGP was dissolved in 100 mL of deionized water. Then 1 g of ascorbic acid was added, the pH was adjusted to 5 with 10% NaOH/HCl, 3.56 g of FeCl_2_·4H_2_O was added, and the mixture was put into a magnetic stirrer to chelate for 40 min, after which the mixture was centrifuged for 1 h at 4000× *g*. Take 1 mL of the supernatant and add the working color development solution according to the procedure of the standard solution, measure the absorbance value at 510 nm, and bring it into the standard curve to calculate the dissolved iron content in the supernatant, in which the chelated iron content of the peptide can be calculated by the formula:

$$\mathrm{Chelated}\;\mathrm{iron}\;\mathrm{content}\;\mathrm{of}\;\mathrm{the}\;\mathrm{peptide}\left(\mu g/\mathrm{mg}\right)=\frac{M1-M2}{M0}$$

M1—added iron content, μg

M2—iron content of supernatant, μg

M0—peptide mass, mg

### 2.5. Chelation of LMW DHGP with Ferrous Chloride

The experimental procedure was performed based on the approach developed by Wang et al. [21], with certain modifications. A 4% mass ratio of LMW DHGP solution was prepared, and then 1% ascorbic acid was added. After pH was adjusted to 5, FeCl_2_·4H_2_O was added with a peptide iron ratio of 4:1. A magnetic stirrer was used to mix the solution at room temperature for 40 min and the mixture was centrifuged at 4000× *g* to separate the solid residue. The supernatant was collected and treated with alcohol precipitation (95% ethanol), and then washed and precipitated with anhydrous ethanol repeatedly. After redissolving the sediment, the LMW DHGP-iron complexes were obtained by freeze-drying at −50 °C for 24 h using a freeze dryer (Labconco, Kansas City, MO, USA).

### 2.6. Scanning Electron Microscopy (SEM) and X-Ray Diffraction (XRD) Analysis of LMW DHGP and LMW DHGP-Iron Complexes

Referring to Chen et al. [22], the microstructure of LMW DHGP freeze-dried powder and LMW DHGP-iron complexes were observed. After the sample was fixed, the vacuum gold spraying was performed, the acceleration voltage was set to 5 kV, and the microstructure of the sample surface was observed using the SEM (Gemini Sigma 300, Zeiss, Germany) at 500×, 5000×, 10,000× and 20,000×, respectively.

The XRD analysis was performed according to the method described by Zhang et al. [23] with modifications. The X-ray diffractometer was operated at 40 kV and 30 mA, with a scanning range of 5–70° (2θ) at a step size of 0.02° and a counting time of 0.3 s per step.

### 2.7. Analysis of the Amino Acid Composition of LMW DHGP and LMW DHGP-Iron Complexes

The Biochrom 30+ amino acid analyzer (Biochrom Ltd., FCE, Cambridge, UK) was used to detect the amino acid composition of LMW DHGP and LMW DHGP-iron complexes. The 100 mg sample was placed in ampoules with 10 mL 6 mol/L HCl or 4 mol/L NaOH. Then, it was sealed by an alcohol blowtorch and hydrolyzed in the oven at 110 °C for 24 h. After the hydrolysis, the sample was transferred to the evaporating dish for drying in a water bath at 80 °C. The evaporating dish was cleaned several times with the derived buffer, after which all the lotion was transferred to the 50 mL volumetric bottle. Then, the sample solution was set volume to scale with the derived buffer and filtered through a 0.45-µm filter. Took out 5 mL solution in 25 mL brown volumetric bottle, added 2.5 mL derivative solution, mixed evenly, and carried out dark reaction under 60 °C water bath for 60 min. After the reaction was finished, it was cooled to room temperature and diluted to scale with an equilibrium buffer. Subsequent to filtration through 0.45-µm membranes, the samples were subjected to instrumental analysis. The buffer and reaction solutions were delivered at flow rates of 20 mL/h and 10 mL/h, respectively. The sample volume was 50 µL, the column temperature was 25 °C, and the detection wavelengths were 570 nm and 440 nm.

### 2.8. UV and FL Analysis of LMW DHGP and LMW DHGP-Iron Complexes

Referring to Yuan et al. [24] and Zheng et al. [25], LMW DHGP and LMW DHGP-iron complexes were dissolved in ultrapure water at a concentration of 1 mg/mL, respectively, and the ultrapure water was used as a blank control. The fluorescence spectroscopy was scanned with a microplate reader at an excitation wavelength of 280 nm and an emission wavelength of 230–500 nm, respectively. While, the UV-Vis spectroscopy of samples was determined by a microplate reader at 200–900 nm.

### 2.9. Fourier Transform Infrared Spectroscopy (FTIR) Analysis of LMW DHGP and LMW DHGP-Iron Complexes

The 2 mg of freeze-dried powder of LMW DHGP or LMW DHGP-iron complexes was mixed with 200 mg dry KBr powder and ground into thin slices, respectively. With KBr as the blank background, the Fourier transform infrared spectrometer (Thermo Nicolet iS5, Thermo Fisher, Waltham, MA, USA) was used for infrared spectrum scanning in the range of 4000–400 cm^−1^ wave number. Each sample was scanned 32 times and FTIR spectra were obtained.

### 2.10. Circular Dichroism Spectroscopy (CD) Analysis of LMW DHGP and LMW DHGP-Iron Complexes

The method described by Ding et al. [26] was utilized with some modifications, the secondary structure of LMW DHGP and LMW DHGP-iron complexes was determined by a circular dichroism spectrometer (Applied Photophysics Ltd., FCE, Surrey, UK). Under the premise of meeting the test voltage, the concentration of the sample was configured as 1 mg/mL. The spectral scanning range was set to 190–270 nm, the scanning speed was 100 nm/min, and the optimal bandwidth was 1 nm. Spectra Analysis software (Circular Dichroism Neural Network) was used to calculate the ellipticity of the test samples, and the secondary structures of LMW DHGP and LMW DHGP-iron complexes were calculated by Reed’s equation.

### 2.11. In Vitro Assessment of the Solubility Stability of LMW DHGP and LMW DHGP-Iron Complexes

The LMW DHGP-iron complexes were subjected to a two-stage simulated gastrointestinal digestion with minor modifications as described by Qu et al. [27]. The simulated gastric digest was prepared by dissolving porcine pepsin (40 mg) in 1 mL of 0.1 N HCl, while the intestinal digest contained trypsin (20 mg) and bile salts (120 mg) in 10 mL of 0.1 M NaHCO_3_. The LMW DHGP-iron complexes and FeSO_4_ solution were stirred and incubated separately in a water bath at 37 °C for 30 min. The pH of the reaction solution was adjusted to 2.0, and then the prepared simulated gastric digest was added so that the final enzyme/substrate ratio was 1:100 (*w*/*w*). Samples were taken at 0, 30, 60, 90, and 120 min of the simulated gastric digestion. After 120 min of gastric digestion, the pH was adjusted to 7.5 and then freshly prepared mock enteric digest was added to the reaction solution. Samples were taken at 0, 30, 60, 90, and 120 min of the simulated enteric digestion to be measured. At the termination phase of the simulated GI tract digestion, reaction solution was heated to 80 °C and maintained for 20 min to terminate the reaction. The samples collected during the experiment were centrifuged at 10,000× *g* for 10 min at 4 °C, and the supernatant was collected, and the solubility of ferrous ions was calculated as follows:

$$\mathrm{Iron}\;\mathrm{ion}\;\mathrm{solubility}\left(\%\right)=\frac{A}{B}\times 100\%$$

A—iron content in supernatant

B—total iron in solution

### 2.12. Determination of the Antioxidant Activity

Previous studies had isolated 26 pure pentapeptides from LMW DHGP, among which the five peptides demonstrating the strongest iron-chelating capacity were selected. The DPPH and ABTS radical scavenging activities of these pentapeptides, along with LMW DHGP and their chelates, were determined according to the method described by Liang et al. [28] with minor modifications.

A mixture of 100 μL sample solution (5 mg/mL), 100 μL freshly prepared DPPH solution (0.6 mM in methanol), and 100 μL methanol was added to a 96-well microplate. Methanol served as the blank control. The reaction was carried out in the dark at room temperature for 30 min, after which the absorbance was measured at 517 nm using a microplate reader. The DPPH radical scavenging activity was calculated using the following formula:

$$\mathrm{DPPH}\;\mathrm{radical}\;\mathrm{scavening}\;\mathrm{activity}\;\left(\%\right)=\left(1-\frac{\mathrm{Asample}}{\mathrm{Ablank}}\right)\times 100\%$$

A mixture of ABTS (5 mL, 7 mM) and potassium persulfate (5 mL, 2.45 mM) solutions was prepared and incubated in the dark for 16 h. The resulting solution was diluted with deionized water until an absorbance of 0.70 ± 0.02 at 734 nm was achieved. The sample solution (5 mg/mL) was combined with diluted ABTS solution at a 1:3 ratio (50 μL:150 μL) in a 96-well microplate. Deionized water served as the blank control. After 6 min of incubation in the dark at room temperature, the absorbance was measured at 734 nm. The ABTS radical scavenging activity was calculated using the following formula:

$$\mathrm{ABTS}\;\mathrm{radical}\;\mathrm{scavening}\;\mathrm{activity}\;\left(\%\right)=\left(1-\frac{\mathrm{Asample}}{\mathrm{Ablank}}\right)\times 100\%$$

### 2.13. Statistical Analysis

Each set of experiments was repeated three times. SPSS Statistics 27.0 statistical analysis software (SPSS Inc., Chicago, IL, USA) was used to conduct a one-way analysis of variance (ANOVA) analysis of the data of each group. Different labeling letters indicated significant differences between the data (*p* < 0.05). Experimental data were expressed as mean ± standard deviation.

## 3. Results and Discussion

### 3.1. Molecular Weight Distribution and Iron Chelating Ability of LMW DHGP

**Figure 1:**
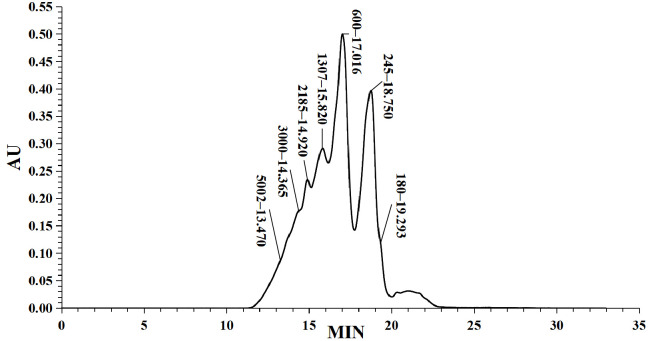

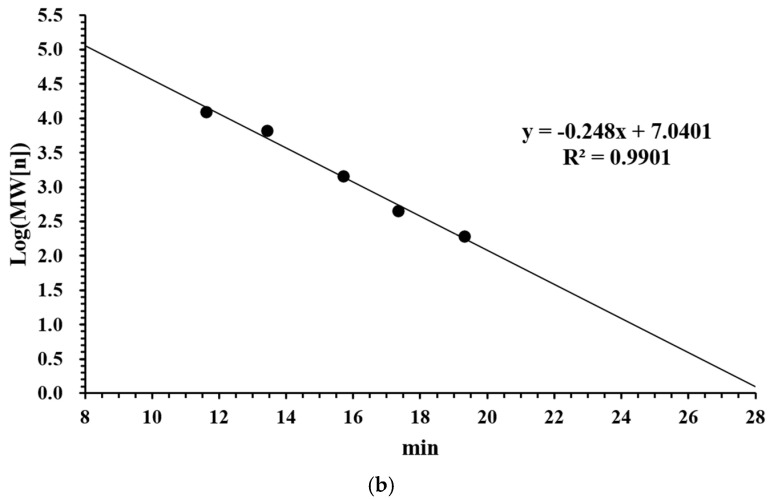
Molecular weight distribution analysis of LMW DHGP. (**a**) Molecular weight distribution, (**b**) Molecular weight calibration curve.

In this study, the iron ion binding capacity of LMW DHGP was determined to be 249.98 μg/mg. Previous studies have demonstrated that the molecular weight of peptides significantly influences their iron ion chelating ability, with higher chelating capacity predominantly observed in small molecular weight peptides [20]. The molecular weight distribution of LMW DHGP was analyzed using high-performance liquid chromatography (HPLC), which was illustrated in Figure 1. The chromatographic separation revealed distinct retention times corresponding to different molecular weight fractions. The standard curve was established as: y = −0.248x + 7.0401, with a correlation coefficient (R^2^) of 0.9901. The molecular weight distribution of LMW DHGP was categorized into eight fractions: >10,000 Da (4.89%), 10,000–5000 Da (7.56%), 5000–3000 Da (8.70%), 3000–2000 Da (18.91%), 2000–1000 Da (27.41%), 1000–500 Da (26.66%), 500–180 Da (5.86%), and <180 Da (4.89%). The average molecular weight of the sample was calculated to be 1401 Da. Notably, 83.73% of the fractions exhibited molecular weights below 3000 Da, indicating that the majority of LMW DHGP components are small molecular weight peptides which exhibit superior antioxidant activity compared to higher molecular weight peptides [28]. These findings are consistent with the iron chelation experimental results reported by Lin et al. in their study on tilapia skin collagen hydrolysate [29].

### 3.2. Morphological Characterization and X-Ray Diffraction of LMW DHGP and LMW DHGP-Iron Complexes

**Figure 2:**
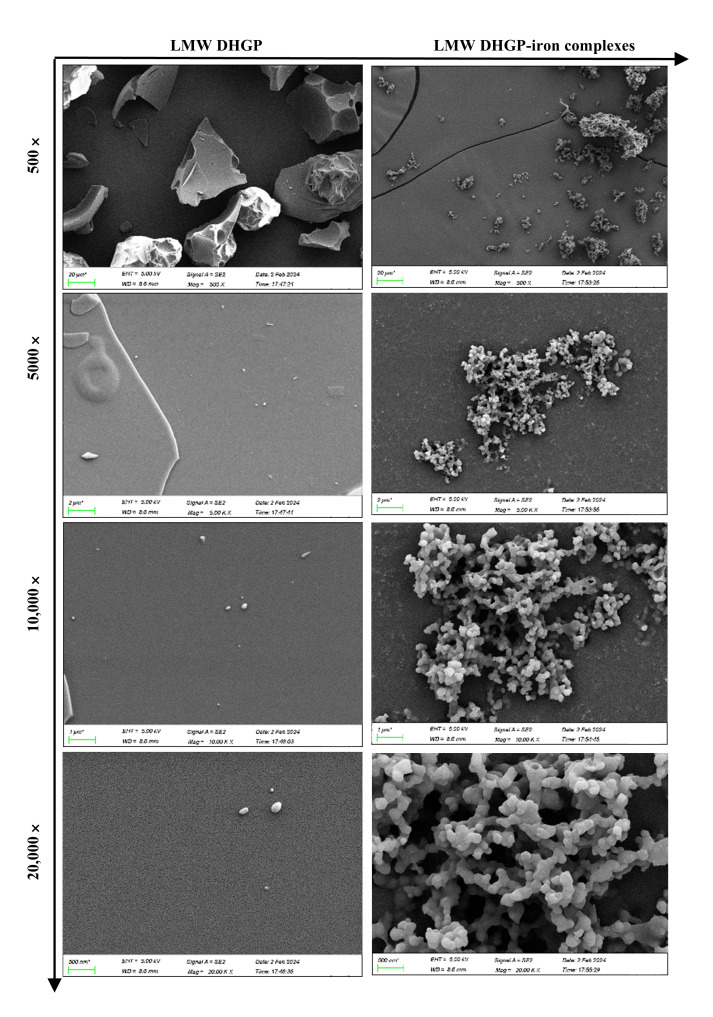
SEM of LMW DHGP and LMW DHGP-iron complexes at 500×, 5000×, 10,000×, and 20,000×.

The morphological characteristics of LMW DHGP and LMW DHGP-iron complexes were investigated using scanning electron microscopy (SEM) at various magnifications (500×, 5000×, 10,000×, and 20,000×). As depicted in Figure 2, distinct differences in size, shape, and surface features were observed between LMW DHGP and LMW DHGP-iron complexes. At a 500× magnification, the surface of LMW DHGP exhibited a smooth and lamellar structure, whereas the LMW DHGP-iron complexes displayed numerous smaller, irregularly shaped particles with some minor convex formations. Upon increasing the magnification to 5000× and 10,000×, the LMW DHGP surface appeared nearly smooth and planar, in contrast to the LMW DHGP-iron complexes, which formed globular particles that aggregated into clusters. At the highest magnification of 20,000×, the spherical particles on the surface of the LMW DHGP-iron complexes were distinctly visible, exhibiting more folds and crystalline structures compared to the LMW DHGP.

In summary, the surface of LMW DHGP was smooth and dense, while that of the LMW DHGP-iron complexes was rough and porous. This transformation suggests that the chelation of ferrous ions altered the originally smooth and loose structure of LMW DHGP into a more compact, aggregated particulate form. Specifically, ferrous ions induced the aggregation of smaller and medium-sized particles of LMW DHGP, disrupting the plate-like structure and leading to the formation of larger spherical particles. This observation aligns with the findings of fermented scallop skirts hydrolysate-iron complex [20]. The observed microstructural changes are likely attributable to the interactions between iron ions and peptides within the LMW DHGP-iron complexes. These interactions result in the formation of dense nanoparticles, indicating a significant structural reorganization upon iron binding.

**Figure 3:**
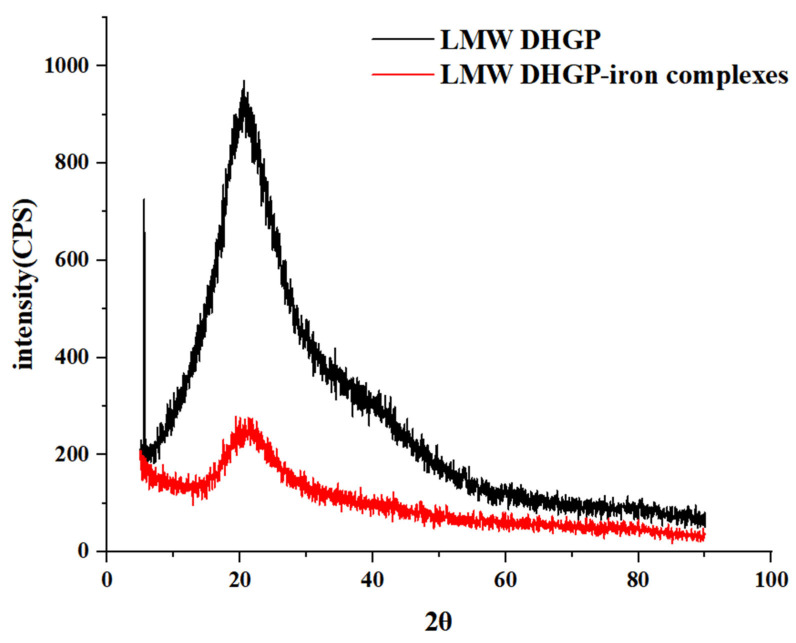
XRD of LMW DHGP and LMW DHGP-iron complexes.

X-ray diffraction (XRD) can be used to investigate the structural changes of various biomacromolecules. As shown in Figure 3, LMW DHGP exhibited a characteristic diffraction peak at 2θ = 5.56°, which disappeared after iron chelation. Additionally, LMW DHGP displayed a distinct diffraction peak at 2θ = 20.6°, the intensity of which decreased with a slight angular shift upon iron binding, indicating the formation of new intermolecular forces and an amorphous structure distinct from that of LMW DHGP. These findings are consistent with previous XRD studies [30,31]. In addition, Qin and Ju [32] reported that the characteristic diffraction peak intensity of fish scale polypeptide significantly increased after iron chelation.

### 3.3. Amino Acid Composition of LMW DHGP and LMW DHGP-Iron Complexes

To identify the amino acids involved in LMW DHGP-iron chelation, we quantitatively analyzed the amino acid composition of both LMW DHGP and its iron complexes, with the detailed results presented in Table 1. The amino acid profiles of LMW DHGP and LMW DHGP-iron complexes were found to be similar, with both being enriched in Gly, Pro, and Glu. However, significant changes in the relative content of certain amino acids were observed during the chelation of LMW DHGP with iron ions. Specifically, the relative content of Asn (N), Gly (G), Cys (C), and Lys (K) increased in the LMW DHGP-iron complexes compared to LMW DHGP, suggesting that these amino acids may play a critical role in the binding of LMW DHGP to iron. Wu et al. [31] conducted iron chelation experiments with whey protein and reported that the levels of Asp, Lys, Glu, Arg, and His were positively correlated with the iron chelation rate, that is, the higher the contents of Asp, Lys, Glu, Arg, and His in whey protein, the greater its iron chelation efficiency. In contrast, Ser levels exhibited a negative correlation. Similarly, after binding sea cucumber peptides to iron, the relative contents of Glu, Asp, and Gly in sea cucumber peptides-iron were significantly increased, with Gly content rising by 3.39%, indicating that these amino acids are crucial for iron chelation [33]. Studies on tilapia skin collagen further highlighted the important role of Lys in iron chelation [29]. In contrast, molecular docking experiments suggested that Lys did not bind to Fe^2+^, likely due to the protonation of Lys at pH 7.0, which is unfavorable for chelation. These discrepancies show that pH is essential for protonation and therefore for chelation of metal ions [26]. In conclusion, the amino acids Asn (N), Gly (G), Cys (C), and Lys (K) are likely involved in the chelation of LMW DHGP with iron, as evidenced by the observed changes in their relative contents.

### 3.4. UV and FL Analysis of LMW DHGP and LMW DHGP-Iron Complexes

**Figure 4:**
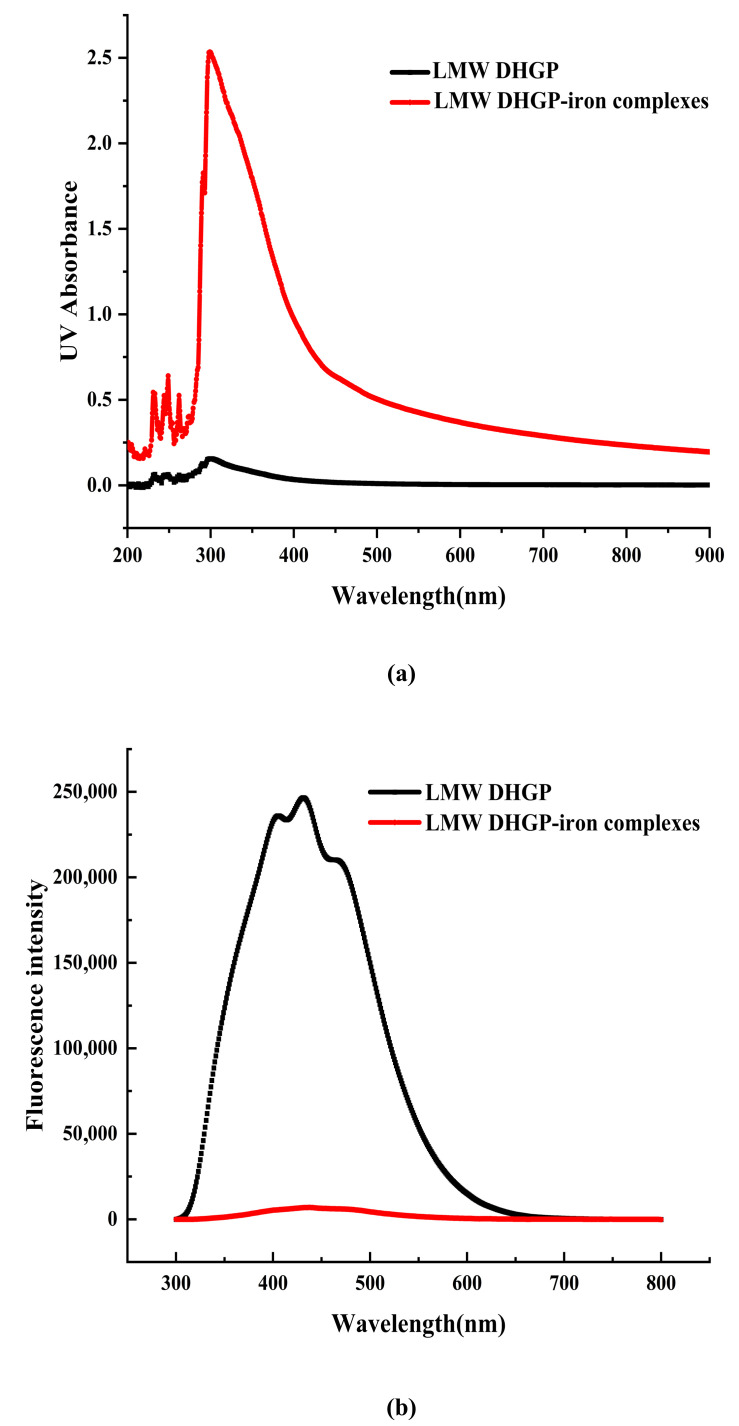
The UV-Vis spectra (**a**) and fluorescence spectrum (**b**) of LMW DHGP and LMW DHGP-iron complexes.

The shift and intensity change of the absorption peak in the UV-Vis spectrum can be used to determine whether LMW DHGP has a chelation reaction with Fe^2+^. As illustrated in Figure 4, the UV-Vis spectra of LMW DHGP and LMW DHGP-iron complexes exhibit significant differences. The maximum absorption peak of LMW DHGP was observed at 301 nm. Upon chelation with iron, the maximum absorption peak of the LMW DHGP-iron complexes shifted to 299 nm, accompanied by an increase in absorbance intensity compared to LMW DHGP. This shift and intensity change suggest that the chelation of LMW DHGP with Fe^2+^ involves electron migration and molecular or atomic interactions [10]. Both LMW DHGP and LMW DHGP-iron complexes displayed a weak absorption peak near 260 nm, which is characteristic of aromatic amino acids [34]. Additionally, LMW DHGP exhibited a weak absorption peak at 276 nm, which disappeared after chelation. This disappearance may be attributed to charge migration involving Tyr, Trp, and Phe upon chelation with iron [11]. A similar phenomenon has been reported in the case of β-casein phosphopeptides-iron chelate [10]. These findings collectively indicate that LMW DHGP undergoes chelation with Fe^2+^, resulting in the formation of a novel complex with distinct spectroscopic properties. The observed spectral changes underscore the significant structural and electronic alterations associated with the chelation process.

The fluorescence spectrum of LMW DHGP exhibits a maximum absorption peak at 431 nm. Upon binding with Fe^2+^, the fluorescence intensity significantly decreases, which is similar to the fluorescence spectral results observed for the powder of large yellow croaker roes hydrolysates and their iron chelates [35]. Yang et al. [36] also demonstrated that the fluorescence intensity of various components of nucleolar peptides decreases after chelation with iron. Similarly, the fluorescence intensity of chicken blood peptide markedly decreases upon chelation with iron [34]. These results indicate that the chelation of Fe^2+^ with LMW DHGP alters the peptide’s structure, leading to fluorescence quenching. This suggests that the fluorescent residues are less exposed to the solvent, resulting in a reduction in fluorescence intensity. Consequently, it can be concluded that LMW DHGP forms a new substance upon chelation with Fe^2+^.

### 3.5. Results of FTIR of LMW DHGP and LMW DHGP-Iron Complexes

**Figure 5:**
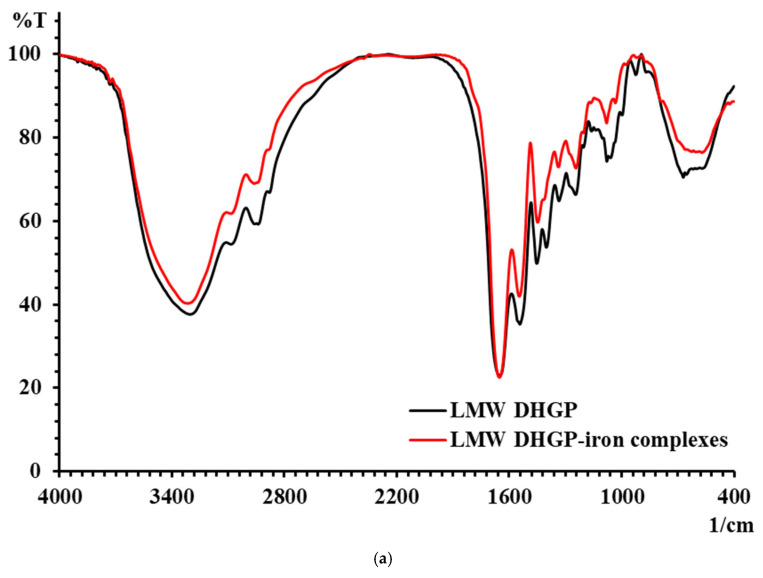

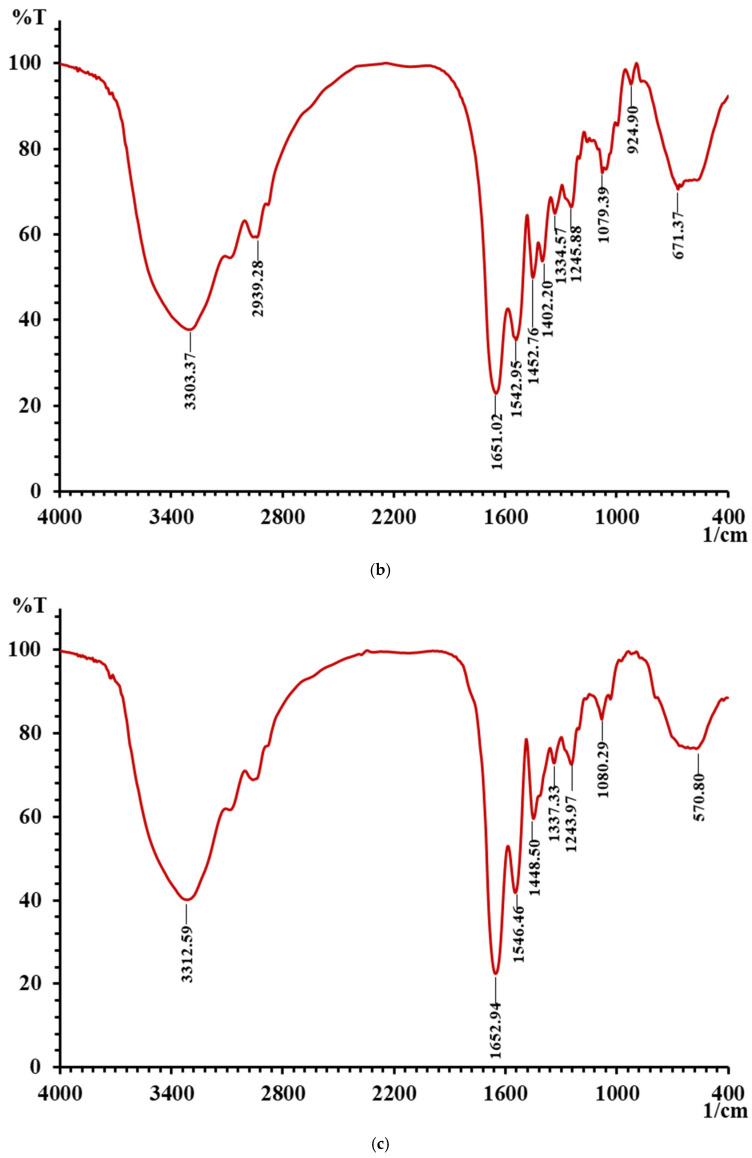
(**a**) The FTIR spectra of LMW DHGP and LMW DHGP-iron complexes, (**b**) the FTIR spectra of LMW DHGP, and (**c**) the FTIR spectra of LMW DHGP-iron complexes.

The changes in characteristic absorption peaks in FTIR spectra can effectively reflect the interactions between metal ions and organic ligands. The FTIR spectra of LMW DHGP and LMW DHGP-iron complexes, as shown in Figure 5, reveal significant differences, indicating that Fe^2+^ binds to specific functional groups within LMW DHGP. The absorption peak of LMW DHGP shifted from 3303.37 cm^−1^ to 3312.59 cm^−1^, corresponding to the stretching vibration of the N-H bond. This shift suggests the replacement of the N-H bond by an N-Fe bond upon chelation. The absorption peak at 1651.02 cm^−1^, consistent with the amide I band (1700–1600 cm^−1^), is associated with the stretching vibration of the C=O bond. After chelation, the absorption peaks of LMW DHGP shifted from 1651.02 cm^−1^ and 1452.76 cm^−1^ to 1652.94 cm^−1^ and 1448.50 cm^−1^, respectively. This shift indicates that the -COOH group reacts with Fe^2+^ to form -COO-Fe complexes [10,14]. The absorption peak at 1542.94 cm^−1^, corresponding to the amide II band (1600–1500 cm^−1^), is related to the bending vibration of the N-H bond. Upon chelation with iron, this peak shifted to 1546.46 cm^−1^, likely due to the replacement of hydrogen in the N-H bond by Fe^2+^, resulting in the formation of a Fe-N bond. Additionally, the intensity of the absorption peak at 1080.29 cm^−1^ in LMW DHGP-iron complexes was higher than that of LMW DHGP at 1079.39 cm^−1^, which can be attributed to the interaction between amino groups and iron [21].

In summary, the chelation of LMW DHGP with iron to form LMW DHGP-iron complexes led to significant changes in the positions and relative intensities of characteristic absorption peaks, confirming the formation of a new substance distinct from the original peptide. The primary chelation sites of LMW DHGP with iron involve amino nitrogen and carboxy oxygen, consistent with findings from studies on walnut peptide-iron chelates [37] and iron chelates of silver carp scales [38]. Combined with amino acid analysis and FTIR spectroscopy results, it is evident that LMW DHGP interacts with Fe^2+^ through carboxyl and amino groups of Asn, Gly, Cys, and Lys, forming stable LMW DHGP-iron complexes.

### 3.6. Secondary Structure of LMW DHGP and LMW DHGP-Iron Complexes

**Figure 6:**
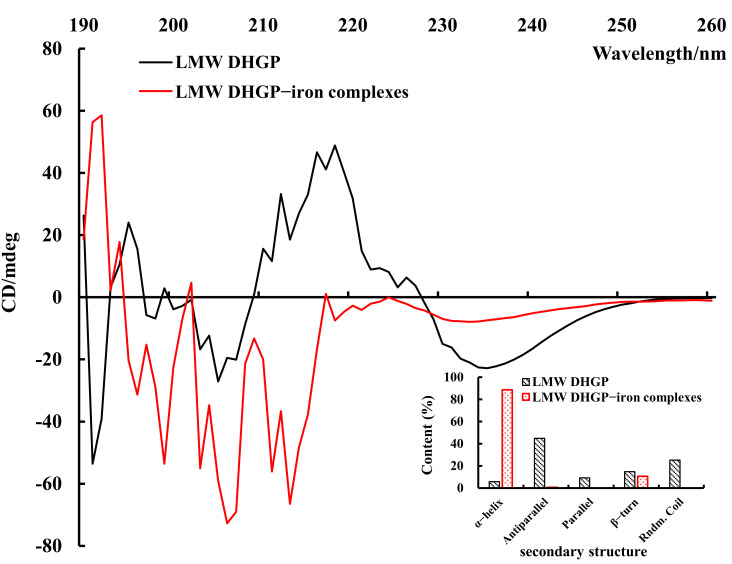
Secondary structure results of LMW DHGP and LMW DHGP-iron complexes. Figure 6 presents the circular dichroism (CD) spectrum along with the derived secondary structure content. The CD spectroscopy wavelength was set as 190–270 nm and the scanning speed was 100 nm/min for three accumulations.

The self-assembly behavior of polypeptides through metal coordination is often accompanied by changes in their secondary structure. Circular dichroism (CD) spectroscopic analysis serves as a principal method for characterizing such structural alterations in proteins or peptides [31]. As illustrated in Figure 6, significant differences are observed in the CD spectra of LMW DHGP-iron complexes compared to those of LMW DHGP alone. Specifically, LMW DHGP exhibits two prominent negative peaks at 200–210 nm and 230–240 nm, whereas the LMW DHGP-iron complexes display a larger negative peak at 210–220 nm. Notably, the peak intensity at 230–240 nm is significantly reduced, and a negative peak emerges at 210–220 nm for the LMW DHGP-iron complexes. These spectral changes indicate substantial alterations in the secondary structure during the formation of LMW DHGP-iron complexes. Quantitative analysis of the secondary structure content revealed that, compared to LMW DHGP, the α-helix content of LMW DHGP-iron complexes increased significantly by 82.99%, while the antiparallel content decreased by 44.25%, and the β-turn structure decreased by 4.2%. Additionally, the parallel and random coil structures were no longer detectable. These results suggest that the formation of LMW DHGP-iron complexes is likely driven by peptide self-assembly behavior predominantly governed by the α-helix structure, rather than being controlled by the β-sheet structure. This shift in structural dominance highlights the critical role of α-helix formation in the metal coordination-driven self-assembly process.

### 3.7. In Vitro Assessment of the Solubility Stability of LMW DHGP and LMW DHGP-Iron Complexes

Peptide assembly behavior dominated by β-sheet structures is typically rigid and highly stable, whereas α-helical assembly exhibits a degree of reversibility. The latter is more susceptible to environmental factors such as temperature and pH. Additionally, when iron preparations are administered orally, their solubility and bioavailability may be compromised due to interactions with digestive enzymes and dietary components such as oxalic acid in the gastrointestinal tract [39]. Therefore, evaluating the solubility of LMW DHGP-iron complexes under digestive conditions is crucial.

**Figure 7:**
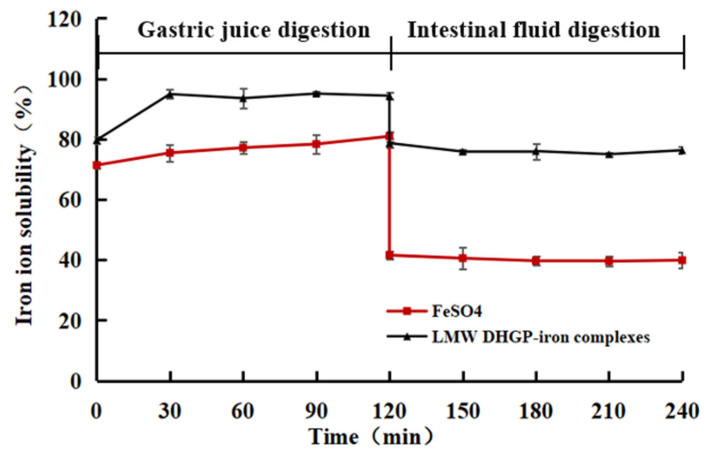
In vitro assessment of the solubility stability of LMW DHGP-iron complexes.

In this study, the solubility of iron ions in LMW DHGP-iron complexes was investigated under simulated gastrointestinal conditions using in vitro digestion experiments. FeSO_4_ served as a negative control, while LMW DHGP-iron complexes were used as a positive control. As shown in Figure 7, the iron ion solubility of LMW DHGP-iron complexes was consistently higher than that of FeSO_4_ during both simulated gastric (pH = 2) and intestinal (pH = 7) digestion phases. During the simulated gastric digestion stage, the iron ion solubility of both LMW DHGP-iron complexes and FeSO_4_ exceeded 70%. However, during the simulated intestinal digestion stage, the iron ion solubility of both decreased, with LMW DHGP-iron complexes maintaining approximately 70% solubility, while FeSO_4_ dropped to around 40%. This phenomenon can be attributed to the strongly acidic environment of gastric juice, where an abundance of H^+^ ions competes with Fe^2+^ for binding sites on LMW DHGP. Consequently, Fe^2+^ readily dissociates from LMW DHGP-iron complexes, leading to a higher iron release rate. In contrast, at intestinal pH values, the deprotonation of -COOH to -COO- and the increased availability of -NH_2_ groups are promoted, facilitating the re-chelating of free Fe^2+^ with the peptide. This results in a lower overall iron release rate in the system [38]. These findings align with those of Zhang et al. [40] and Wu et al. [31], demonstrating that LMW DHGP-iron complexes enhance the solubility of iron ions under simulated gastrointestinal conditions.

### 3.8. Determination of the Antioxidant Activity

**Figure 8:**
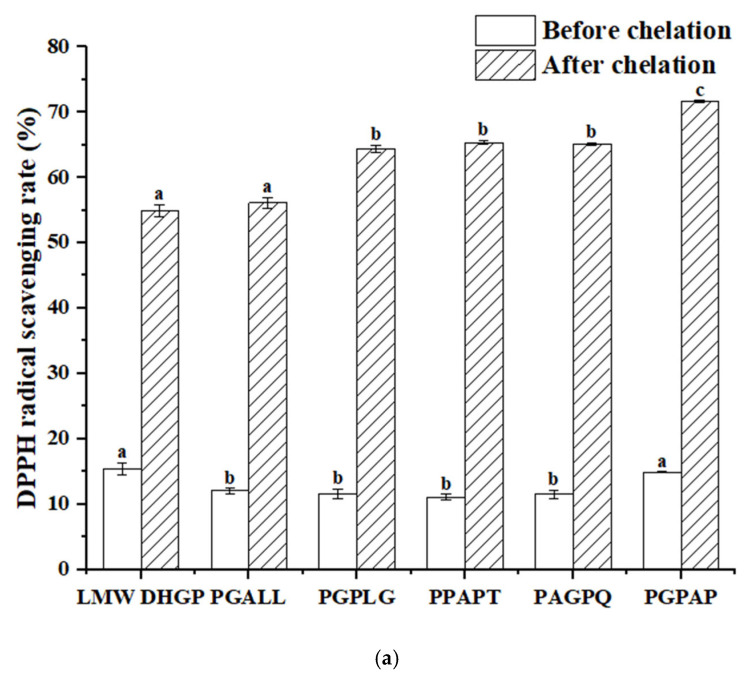

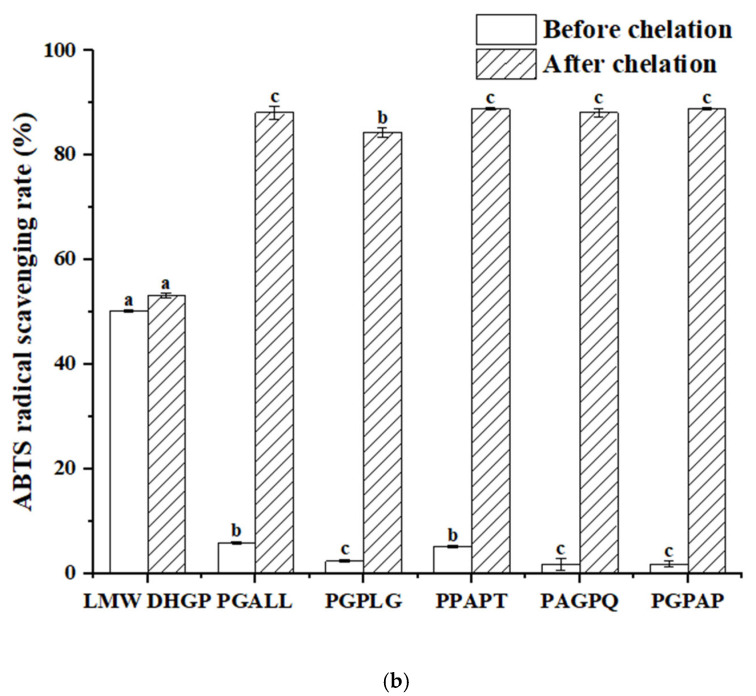
Determination of antioxidant activity of LMW DHGP, pentapeptides, and their chelates. (**a**) DPPH radical scavenging rate of LMW DHGP, pentapeptides, and their chelates, (**b**) ABTS radical scavenging rate of LMW DHGP, pentapeptides, and their chelates. The same letter (a-c) means that the variance of two samples is not significant (*p* > 0.05), and the different letters (a-c) mean significant (*p* < 0.05).

The antioxidant activity of peptides is a critical indicator for screening functional peptides. In this study, the five peptides with the highest iron-chelating activity identified from LMW DHGP were compared with LMW DHGP. As illustrated in Figure 8, LMW DHGP exhibited significantly higher DPPH and ABTS radical scavenging abilities than the other five pentapeptide. Notably, after iron chelation, both LMW DHGP and the pentapeptide demonstrated enhanced antioxidant capacity. The DPPH scavenging rates of LMW DHGP and the pentapeptide were below 16%, whereas their iron-chelated forms showed a remarkable increase to over 54%. Similarly, the ABTS scavenging rates of the purified peptides were less than 5%, but their chelated counterparts achieved over 84%, with PGPAG-iron complexes displaying the most potent antioxidant activity-71.64% (DPPH) and 88.79% (ABTS). These findings indicate that both LMW DHGP and the five peptides exhibit improved antioxidant capacity upon iron chelation, with the purified peptides demonstrating a more pronounced enhancement in radical scavenging compared to LMW DHGP. This highlights the potential of screening specific peptide sequences from LMW DHGP for the development of antioxidant peptide-iron complexes. Wampee seed peptides [14] and Yanbian cattle bone peptides [41] also showed enhanced antioxidant properties after iron chelation. Fan et al. [37] reported that walnut peptide-iron complexes exhibited significantly lower IC50 values for DPPH (3.72 mg/mL) and hydroxyl radical (7.12 mg/mL) scavenging compared to unchelated walnut peptide (4.43 mg/mL and 8.70 mg/mL, respectively). Furthermore, the radical scavenging capacity (DPPH and ABTS) increased proportionally with sample concentration.

### 3.9. Prediction of the Mechanism of Iron Absorption Promotion

Iron absorption by intestinal cells is essential for its biological effects in the body. Fe^2+^ transport occurs via multiple pathways, including the paracellular route, endocytosis, and divalent metal transporter 1 (DMT1) [5]. Fe^3+^ must first be reduced to Fe^2+^ by reductants such as ascorbate, cytochrome b, or brush-border membrane reductases, as well as dietary reductants and gastrointestinal secretions, before absorption via DMT1 [42]. Unutilized iron in enterocytes is stored in ferritin, while iron needed by the body is exported via basolateral ferroportin 1 (FPN1) and distributed by transferrin. Iron (Fe^2+^ and Fe^3+^) can also form chelates with other molecules, enabling absorption in complexed forms through endocytosis or specific importers, followed by transformation or transport as complexes [3]. Zhao et al. [38] identified three potential pathways for iron-chelating peptide transport: peptide transporters, the paracellular route, and endocytosis, with DMT1 being the primary pathway for LR-Fe chelate transport. However, the precise mechanisms of intestinal absorption for peptide-iron chelates, including transcytosis or paracellular routes, require further investigation. Mouse experiments revealed that the serum iron levels in the group administered the casein hydrolysate peptide-iron complex were significantly higher than those in the ferrous sulfate group (*p* < 0.001). Additionally, the concurrent administration of free peptides did not alter the absorption efficiency of either iron source, indicating that the casein hydrolysate peptide-iron complex is absorbed in its intact form rather than through exchange reactions in the gastrointestinal tract. These findings suggest that this complex holds potential as an iron supplement [43]. The three different doses of oat peptide-iron chelate all demonstrated favorable iron-supplementing effects in IDA model mice without adverse side effects. This may be attributed to the unique structure of oat peptide-iron chelate, which regulates its transport and maintains iron absorption to meet physiological demands [44]. Supplementation with β-lactoglobulin hydrolysate-iron complex prevents hemoglobin reduction and normalizes serum ferritin and transferrin levels in anemic mice. This suggests that iron deficiency anemia (IDA) can be prevented by iron supplementation in the form of β-lactoglobulin hydrolysate-iron complex, which may serve as an effective iron source. However, whether it influences the relative gene expression of DMT-1, Dcytb, and PEPT-1 remains to be elucidated [45].

**Figure 9:**
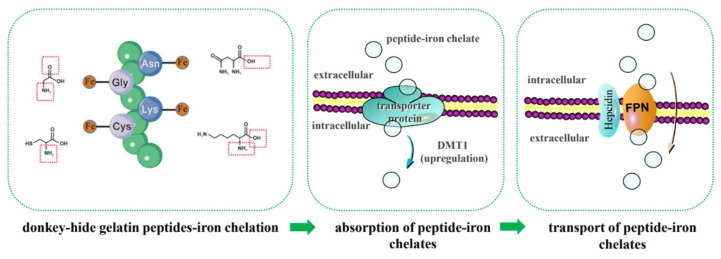
Prediction of iron bioavailability enhancement mechanisms.

Based on the above research background, the following scientific hypotheses are proposed in this study: (1) LMW DHGP binds ferrous ions through coordination with the carboxyl or amino groups of Asn, Gly, Cys, and Lys, thereby enhancing its peptide-ferrous chelating rate and stability; (2) the LMW DHGP-iron complexes primarily enter small intestinal cells via the active transport pathway. The upregulation of FPN1 expression not only promotes the exocytosis of cellular iron but also enhances iron absorption. Furthermore, the upregulation of FPN1 expression may regulate the expression of DMT1 in the ferrous ion uptake pathway, further facilitating iron absorption. The mechanism of iron absorption promotion by small molecule donkey-hide gelatin peptide-iron chelates is predicted as shown in Figure 9. This is the focus of our future research.

## 4. Conclusions

In summary, the iron-binding capacity of LMW DHGP was determined to be 249.98 μg/mg. The chelation of LMW DHGP with iron forms novel complexes, likely driven by α-helix-mediated peptide self-assembly, with potential binding sites at the carboxyl and amino groups of Asn, Gly, Cys, and Lys. The chelation of iron ions disrupted the original lamellar structure of LMW DHGP, leading to the formation of larger spherical particles with a rougher and more porous microstructure. In vitro simulated gastrointestinal digestion experiments demonstrated that the chelation of LMW DHGP with iron significantly enhanced the solubility of iron ions in the simulated gastrointestinal environment, thereby improving iron absorption efficiency. The iron-chelated peptide complexes demonstrated significantly enhanced antioxidant capacity. Notably, PGPAP-iron complexes exhibited superior DPPH and ABTS radical scavenging activities compared to both others pentapeptides-iron and LMW DHGP-iron complexes. Therefore, the LMW DHGP-iron complexes demonstrates dual functionality as a novel iron supplement, exhibiting both enhanced iron bioavailability and significant antioxidant activity. However, the precise mechanism of action still requires further investigation. Additionally, future studies should include cell-based experiments and in vivo research to systematically evaluate the toxicity and iron absorption efficiency of the LMW DHGP-iron complexes, as well as to explore its delivery mechanisms in vivo. These studies will provide a comprehensive assessment of the safety and efficacy of the LMW DHGP-iron complexes as an iron supplement. Furthermore, this research will offer important theoretical foundations and practical guidance for the potential application of the LMW DHGP-iron complexes.

## Figures and Tables

**Table 1:** Amino acid composition of LMW DHGP and LMW DHGP-iron complexes.

NO.	Amino Acid Type	Amino Acid Content of Donkey-Hide Gelatin Peptides (mg/g)	
		LMW DHGP	LMW DHGP-Iron Complexes
1	Asn (N)	54.40	59.61
2	Thr (T)	18.14	14.24
3	Ser (S)	34.67	29.18
4	Glu (E)	96.13	95.40
5	Gly (G)	127.91	128.24
6	Ala (A)	72.32	54.26
7	Cys (C)	16.96	21.37
8	Val (V)	24.85	18.20
9	Met (M)	9.49	7.73
10	Ile (I)	14.08	8.38
11	Leu (L)	30.43	18.43
12	Tyr (Y)	9.18	8.60
13	Phe (F)	19.68	13.22
14	His (H)	7.74	7.70
15	Lys (K)	38.54	39.07
16	Arg (R)	64.85	59.80
17	Pro (P)	110.37	80.41

## Data Availability

The original contributions presented in the study are included in the article, further inquiries can be directed to the corresponding author.

## Abbreviations

LMW	Low molecular weight
DHGP	Donkey-hide gelatin peptide
FTIR	Fourier transform infrared
CD	Circular dichroism
SEM	Scanning electron microscopy
XRD	X-ray diffraction
FL	Fluorescence
